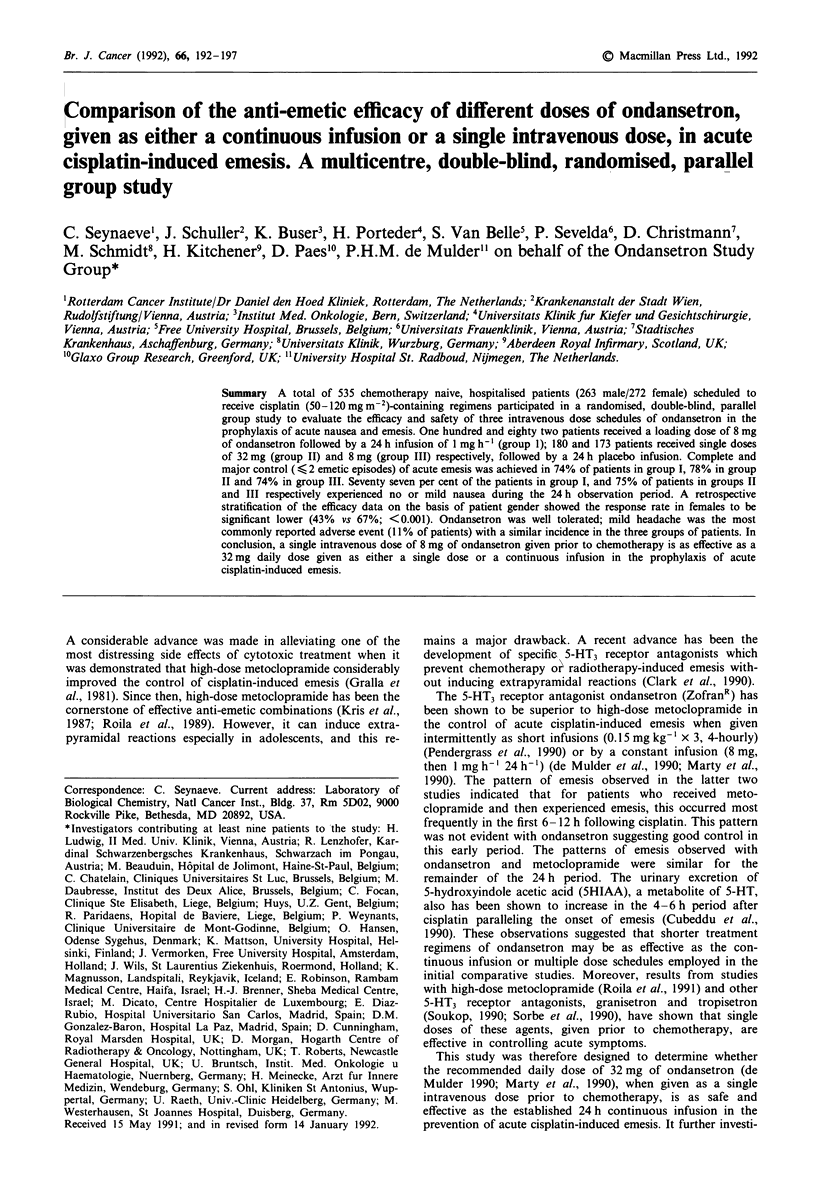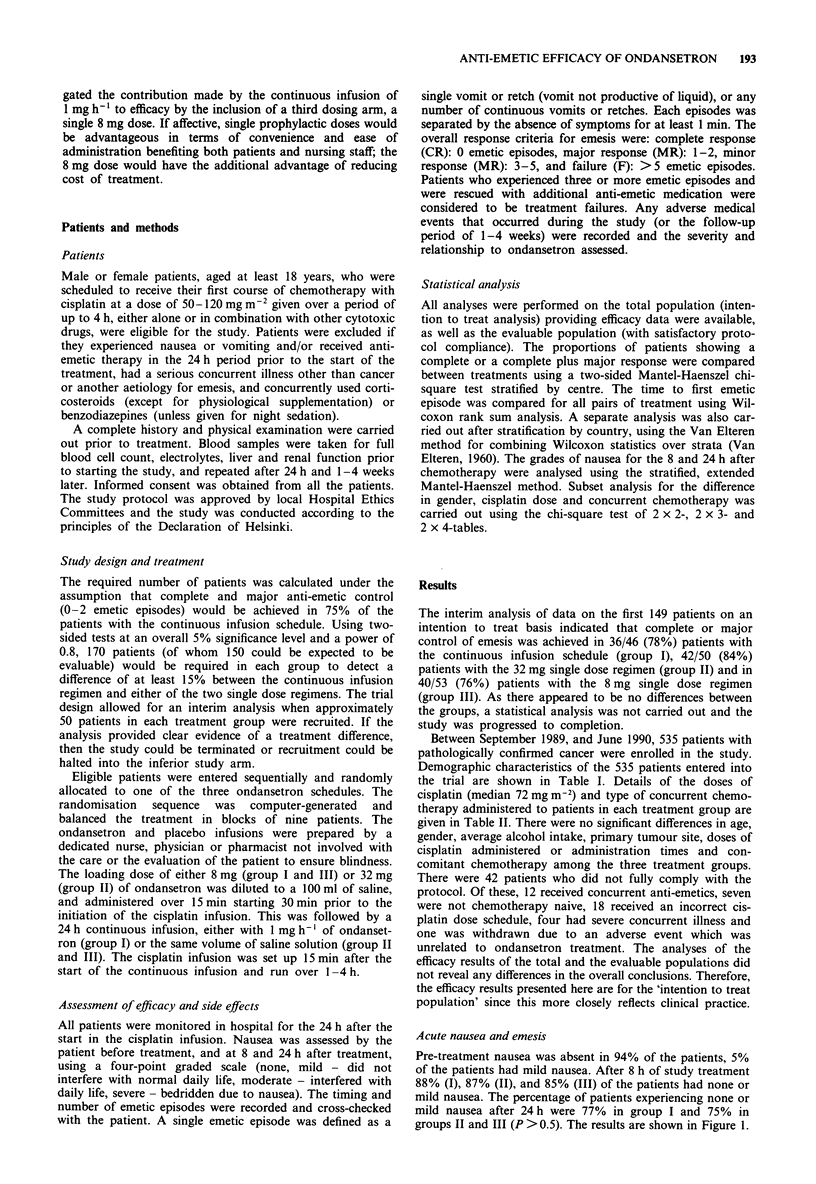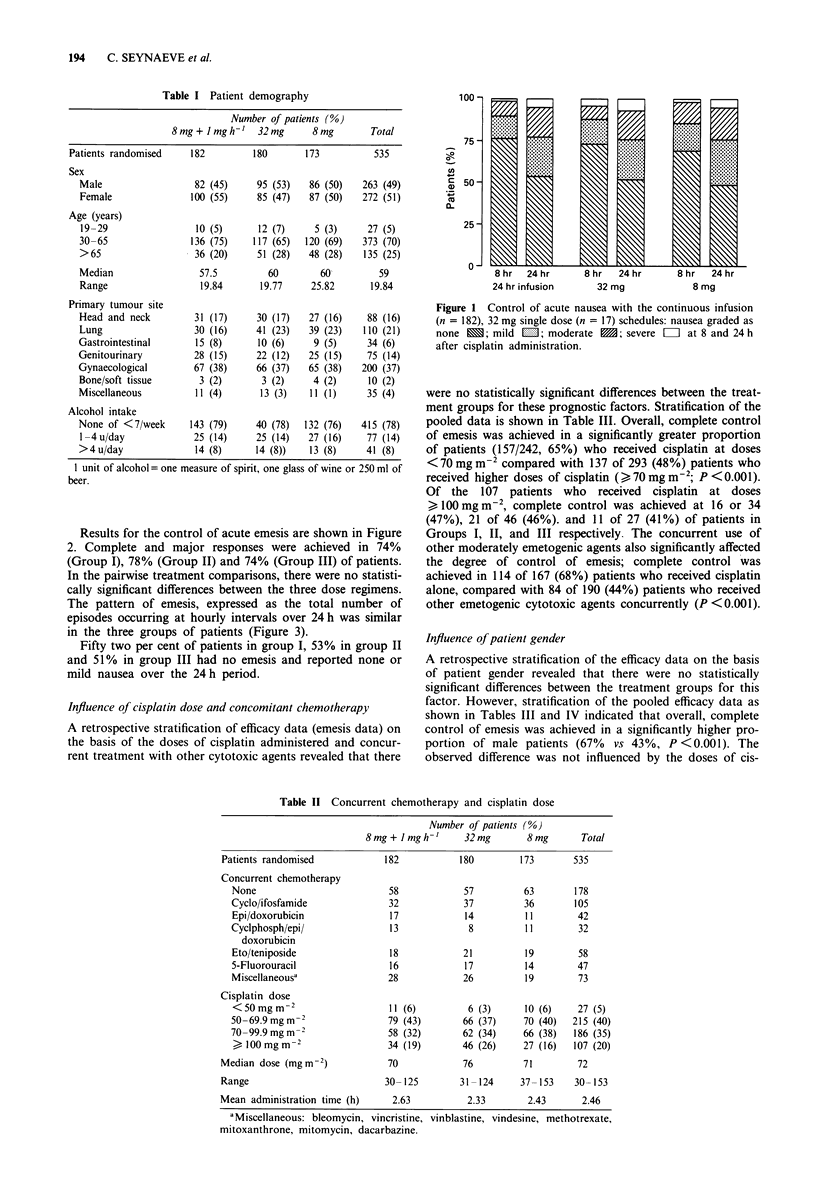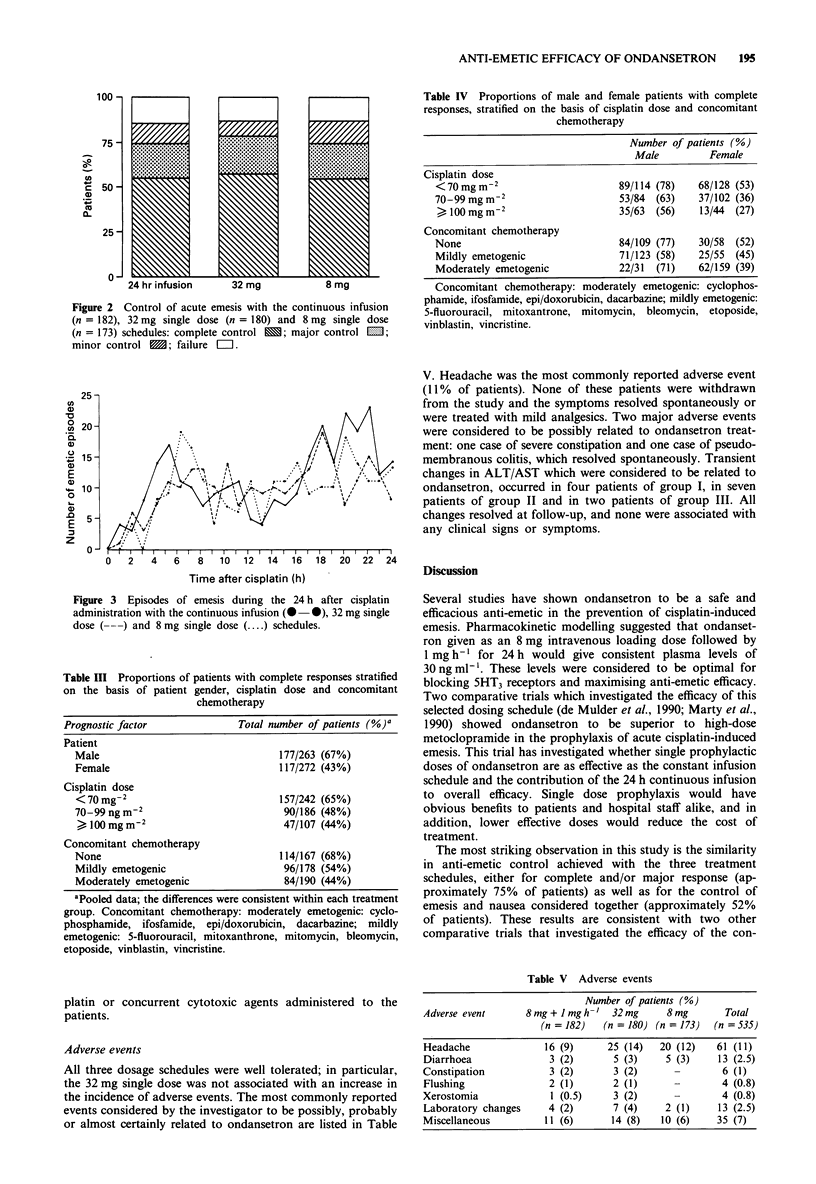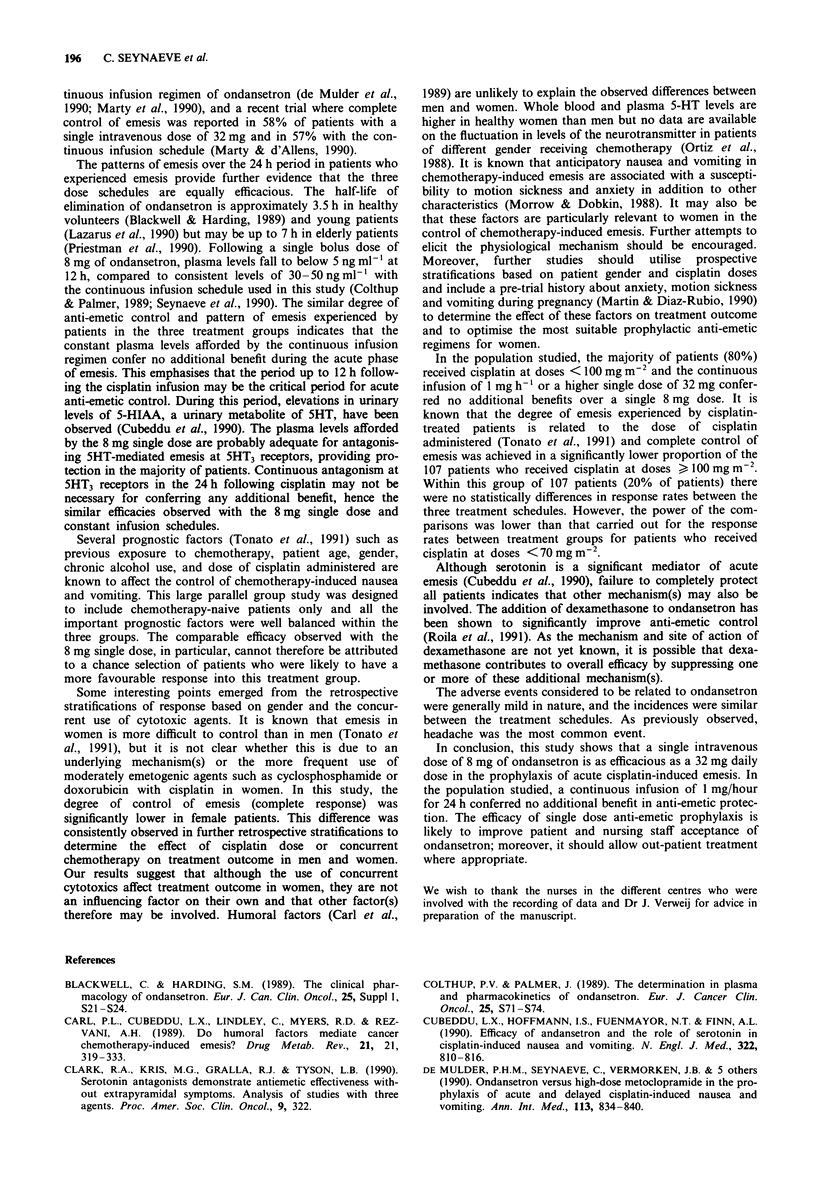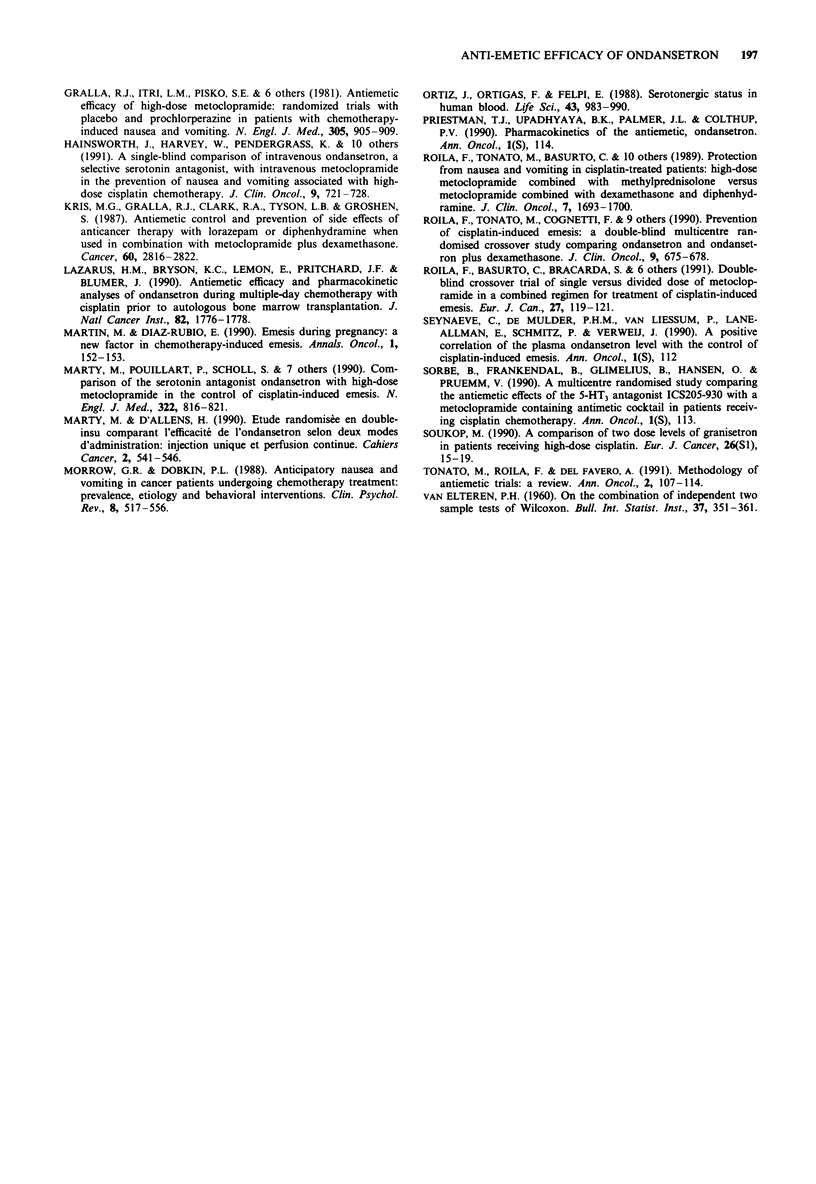# Comparison of the anti-emetic efficacy of different doses of ondansetron, given as either a continuous infusion or a single intravenous dose, in acute cisplatin-induced emesis. A multicentre, double-blind, randomised, parallel group study. Ondansetron Study Group.

**DOI:** 10.1038/bjc.1992.241

**Published:** 1992-07

**Authors:** C. Seynaeve, J. Schuller, K. Buser, H. Porteder, S. Van Belle, P. Sevelda, D. Christmann, M. Schmidt, H. Kitchener, D. Paes

**Affiliations:** Rotterdam Cancer Institute/Dr Daniel den Hoed Kliniek, The Netherlands.

## Abstract

A total of 535 chemotherapy naive, hospitalised patients (263 male/272 female) scheduled to receive cisplatin (50-120 mg m-2)-containing regimens participated in a randomised, double-blind, parallel group study to evaluate the efficacy and safety of three intravenous dose schedules of ondansetron in the prophylaxis of acute nausea and emesis. One hundred and eighty two patients received a loading dose of 8 mg of ondansetron followed by a 24 h infusion of 1 mg h-1 (group 1); 180 and 173 patients received single doses of 32 mg (group II) and 8 mg (group III) respectively, followed by a 24 h placebo infusion. Complete and major control (less than or equal to 2 emetic episodes) of acute emesis was achieved in 74% of patients in group I, 78% in group II and 74% in group III. Seventy seven per cent of the patients in group I, and 75% of patients in groups II and III respectively experienced no or mild nausea during the 24 h observation period. A retrospective stratification of the efficacy data on the basis of patient gender showed the response rate in females to be significant lower (43% vs 67%; less than 0.001). Ondanestron was well tolerated; mild headache was the most commonly reported adverse event (11% of patients) with a similar incidence in the three groups of patients. In conclusion, a single intravenous dose of 8 mg of ondansetron given prior to chemotherapy is as effective as a 32 mg daily dose given as either a single dose of a continuous infusion in the prophylaxis of acute cisplatin-induced emesis.


*Br. J. Cancer* (1992), **66**, 192–197 © Macmillan Press Ltd., 1992

### Comparison of the anti-emetic efficacy of different doses of ondansetron, given as either a continuous infusion or a single intravenous dose, in acute cisplatin-induced emesis. A multicentre, double-blind, randomised, parallel group study

C. Seynaeve[1], J. Schuller[2], K. Buser[3], H. Porteder[4], S. Van Belle[5], P. Sevelda[6], D. Christmann[7], M. Schmidt[8], H. Kitchener[9], D. Paes[10], P.H.M. de Mulder[11] on behalf of the Ondansetron Study Group*

[1]*Rotterdam Cancer Institute/Dr Daniel den Hoed Kliniek, Rotterdam, The Netherlands;* [2]*Krankenanstalt der Stadt Wien, Rudolfstiftung/Vienna, Austria;* [3]*Institut Med. Onkologie, Bern, Switzerland;* [4]*Universitats Klinik fur Kiefer und Gesichtschirurgie, Vienna, Austria;* [5]*Free University Hospital, Brussels, Belgium;* [6]*Universitats Frauenklinik, Vienna, Austria;* [7]*Stadtisches Krankenhaus, Aschaffenburg, Germany;* [8]*Universitats Klinik, Wurzburg, Germany;* [9]*Aberdeen Royal Infirmary, Scotland, UK;* [10]*Glaxo Group Research, Greenford, UK;* [11]*University Hospital St. Radboud, Nijmegen, The Netherlands.*

**Summary** A total of 535 chemotherapy naive, hospitalised patients (263 male/272 female) scheduled to receive cisplatin (50–120 mg m$^{-2}$)-containing regimens participated in a randomised, double-blind, parallel group study to evaluate the efficacy and safety of three intravenous dose schedules of ondansetron in the prophylaxis of acute nausea and emesis. One hundred and eighty two patients received a loading dose of 8 mg of ondansetron followed by a 24 h infusion of 1 mg h$^{-1}$ (group 1); 180 and 173 patients received single doses of 32 mg (group II) and 8 mg (group III) respectively, followed by a 24 h placebo infusion. Complete and major control ($\leqslant 2$ emetic episodes) of acute emesis was achieved in 74% of patients in group I, 78% in group II and 74% in group III. Seventy seven per cent of the patients in group I, and 75% of patients in groups II and III respectively experienced no or mild nausea during the 24 h observation period. A retrospective stratification of the efficacy data on the basis of patient gender showed the response rate in females to be significant lower (43% *vs* 67%; $<0.001$). Ondansetron was well tolerated; mild headache was the most commonly reported adverse event (11% of patients) with a similar incidence in the three groups of patients. In conclusion, a single intravenous dose of 8 mg of ondansetron given prior to chemotherapy is as effective as a 32 mg daily dose given as either a single dose or a continuous infusion in the prophylaxis of acute cisplatin-induced emesis.

A considerable advance was made in alleviating one of the most distressing side effects of cytotoxic treatment when it was demonstrated that high-dose metoclopramide considerably improved the control of cisplatin-induced emesis (Gralla *et al.*, 1981). Since then, high-dose metoclopramide has been the cornerstone of effective anti-emetic combinations (Kris *et al.*, 1987; Roila *et al.*, 1989). However, it can induce extra-pyramidal reactions especially in adolescents, and this remains a major drawback. A recent advance has been the development of specific 5-$HT_3$ receptor antagonists which prevent chemotherapy or radiotherapy-induced emesis without inducing extrapyramidal reactions (Clark *et al.*, 1990).

The 5-$HT_3$ receptor antagonist ondansetron (Zofran[R]) has been shown to be superior to high-dose metoclopramide in the control of acute cisplatin-induced emesis when given intermittently as short infusions (0.15 mg kg$^{-1}$ × 3, 4-hourly) (Pendergrass *et al.*, 1990) or by a constant infusion (8 mg, then 1 mg h$^{-1}$ 24 h$^{-1}$) (de Mulder *et al.*, 1990; Marty *et al.*, 1990). The pattern of emesis observed in the latter two studies indicated that for patients who received metoclopramide and then experienced emesis, this occurred most frequently in the first 6–12 h following cisplatin. This pattern was not evident with ondansetron suggesting good control in this early period. The patterns of emesis observed with ondansetron and metoclopramide were similar for the remainder of the 24 h period. The urinary excretion of 5-hydroxyindole acetic acid (5HIAA), a metabolite of 5-HT, also has been shown to increase in the 4–6 h period after cisplatin paralleling the onset of emesis (Cubeddu *et al.*, 1990). These observations suggested that shorter treatment regimens of ondansetron may be as effective as the continuous infusion or multiple dose schedules employed in the initial comparative studies. Moreover, results from studies with high-dose metoclopramide (Roila *et al.*, 1991) and other 5-$HT_3$ receptor antagonists, granisetron and tropisetron (Soukop, 1990; Sorbe *et al.*, 1990), have shown that single doses of these agents, given prior to chemotherapy, are effective in controlling acute symptoms.

This study was therefore designed to determine whether the recommended daily dose of 32 mg of ondansetron (de Mulder 1990; Marty *et al.*, 1990), when given as a single intravenous dose prior to chemotherapy, is as safe and effective as the established 24 h continuous infusion in the prevention of acute cisplatin-induced emesis. It further investi-

Correspondence: C. Seynaeve. Current address: Laboratory of Biological Chemistry, Natl Cancer Inst., Bldg. 37, Rm 5D02, 9000 Rockville Pike, Bethesda, MD 20892, USA.

*Investigators contributing at least nine patients to the study: H. Ludwig, II Med. Univ. Klinik, Vienna, Austria; R. Lenzhofer, Kardinal Schwarzenbergsches Krankenhaus, Schwarzach im Pongau, Austria; M. Beauduin, Hôpital de Jolimont, Haine-St-Paul, Belgium; C. Chatelain, Cliniques Universitaires St Luc, Brussels, Belgium; M. Daubresse, Institut des Deux Alice, Brussels, Belgium; C. Focan, Clinique Ste Elisabeth, Liege, Belgium; Huys, U.Z. Gent, Belgium; R. Paridaens, Hopital de Baviere, Liege, Belgium; P. Weynants, Clinique Universitaire de Mont-Godinne, Belgium; O. Hansen, Odense Sygehus, Denmark; K. Mattson, University Hospital, Helsinki, Finland; J. Vermorken, Free University Hospital, Amsterdam, Holland; J. Wils, St Laurentius Ziekenhuis, Roermond, Holland; K. Magnusson, Landspitali, Reykjavik, Iceland; E. Robinson, Rambam Medical Centre, Haifa, Israel; H.-J. Brenner, Sheba Medical Centre, Israel; M. Dicato, Centre Hospitalier de Luxembourg; E. Diaz-Rubio, Hospital Universitario San Carlos, Madrid, Spain; D.M. Gonzalez-Baron, Hospital La Paz, Madrid, Spain; D. Cunningham, Royal Marsden Hospital, UK; D. Morgan, Hogarth Centre of Radiotherapy & Oncology, Nottingham, UK; T. Roberts, Newcastle General Hospital, UK; U. Bruntsch, Instit. Med. Onkologie u Haematologie, Nuernberg, Germany; H. Meinecke, Arzt fur Innere Medizin, Wendeburg, Germany; S. Ohl, Kliniken St Antonius, Wuppertal, Germany; U. Raeth, Univ.-Clinic Heidelberg, Germany; M. Westerhausen, St Joannes Hospital, Duisberg, Germany.

Received 15 May 1991; and in revised form 14 January 1992.

gated the contribution made by the continuous infusion of 1 mg $h^{-1}$ to efficacy by the inclusion of a third dosing arm, a single 8 mg dose. If affective, single prophylactic doses would be advantageous in terms of convenience and ease of administration benefiting both patients and nursing staff; the 8 mg dose would have the additional advantage of reducing cost of treatment.

### Patients and methods

### *Patients*

Male or female patients, aged at least 18 years, who were scheduled to receive their first course of chemotherapy with cisplatin at a dose of 50–120 mg $m^{-2}$ given over a period of up to 4 h, either alone or in combination with other cytotoxic drugs, were eligible for the study. Patients were excluded if they experienced nausea or vomiting and/or received anti-emetic therapy in the 24 h period prior to the start of the treatment, had a serious concurrent illness other than cancer or another aetiology for emesis, and concurrently used corticosteroids (except for physiological supplementation) or benzodiazepines (unless given for night sedation).

A complete history and physical examination were carried out prior to treatment. Blood samples were taken for full blood cell count, electrolytes, liver and renal function prior to starting the study, and repeated after 24 h and 1–4 weeks later. Informed consent was obtained from all the patients. The study protocol was approved by local Hospital Ethics Committees and the study was conducted according to the principles of the Declaration of Helsinki.

### *Study design and treatment*

The required number of patients was calculated under the assumption that complete and major anti-emetic control (0–2 emetic episodes) would be achieved in 75% of the patients with the continuous infusion schedule. Using two-sided tests at an overall 5% significance level and a power of 0.8, 170 patients (of whom 150 could be expected to be evaluable) would be required in each group to detect a difference of at least 15% between the continuous infusion regimen and either of the two single dose regimens. The trial design allowed for an interim analysis when approximately 50 patients in each treatment group were recruited. If the analysis provided clear evidence of a treatment difference, then the study could be terminated or recruitment could be halted into the inferior study arm.

Eligible patients were entered sequentially and randomly allocated to one of the three ondansetron schedules. The randomisation sequence was computer-generated and balanced the treatment in blocks of nine patients. The ondansetron and placebo infusions were prepared by a dedicated nurse, physician or pharmacist not involved with the care or the evaluation of the patient to ensure blindness. The loading dose of either 8 mg (group I and III) or 32 mg (group II) of ondansetron was diluted to a 100 ml of saline, and administered over 15 min starting 30 min prior to the initiation of the cisplatin infusion. This was followed by a 24 h continuous infusion, either with 1 mg $h^{-1}$ of ondansetron (group I) or the same volume of saline solution (group II and III). The cisplatin infusion was set up 15 min after the start of the continuous infusion and run over 1–4 h.

### *Assessment of efficacy and side effects*

All patients were monitored in hospital for the 24 h after the start in the cisplatin infusion. Nausea was assessed by the patient before treatment, and at 8 and 24 h after treatment, using a four-point graded scale (none, mild – did not interfere with normal daily life, moderate – interfered with daily life, severe – bedridden due to nausea). The timing and number of emetic episodes were recorded and cross-checked with the patient. A single emetic episode was defined as a single vomit or retch (vomit not productive of liquid), or any number of continuous vomits or retches. Each episodes was separated by the absence of symptoms for at least 1 min. The overall response criteria for emesis were: complete response (CR): 0 emetic episodes, major response (MR): 1–2, minor response (MR): 3–5, and failure (F): $>5$ emetic episodes. Patients who experienced three or more emetic episodes and were rescued with additional anti-emetic medication were considered to be treatment failures. Any adverse medical events that occurred during the study (or the follow-up period of 1–4 weeks) were recorded and the severity and relationship to ondansetron assessed.

### *Statistical analysis*

All analyses were performed on the total population (intention to treat analysis) providing efficacy data were available, as well as the evaluable population (with satisfactory protocol compliance). The proportions of patients showing a complete or a complete plus major response were compared between treatments using a two-sided Mantel-Haenszel chi-square test stratified by centre. The time to first emetic episode was compared for all pairs of treatment using Wilcoxon rank sum analysis. A separate analysis was also carried out after stratification by country, using the Van Elteren method for combining Wilcoxon statistics over strata (Van Elteren, 1960). The grades of nausea for the 8 and 24 h after chemotherapy were analysed using the stratified, extended Mantel-Haenszel method. Subset analysis for the difference in gender, cisplatin dose and concurrent chemotherapy was carried out using the chi-square test of 2 × 2-, 2 × 3- and 2 × 4-tables.

### Results

The interim analysis of data on the first 149 patients on an intention to treat basis indicated that complete or major control of emesis was achieved in 36/46 (78%) patients with the continuous infusion schedule (group I), 42/50 (84%) patients with the 32 mg single dose regimen (group II) and in 40/53 (76%) patients with the 8 mg single dose regimen (group III). As there appeared to be no differences between the groups, a statistical analysis was not carried out and the study was progressed to completion.

Between September 1989, and June 1990, 535 patients with pathologically confirmed cancer were enrolled in the study. Demographic characteristics of the 535 patients entered into the trial are shown in Table I. Details of the doses of cisplatin (median 72 mg $m^{-2}$) and type of concurrent chemotherapy administered to patients in each treatment group are given in Table II. There were no significant differences in age, gender, average alcohol intake, primary tumour site, doses of cisplatin administered or administration times and concomitant chemotherapy among the three treatment groups. There were 42 patients who did not fully comply with the protocol. Of these, 12 received concurrent anti-emetics, seven were not chemotherapy naive, 18 received an incorrect cisplatin dose schedule, four had severe concurrent illness and one was withdrawn due to an adverse event which was unrelated to ondansetron treatment. The analyses of the efficacy results of the total and the evaluable populations did not reveal any differences in the overall conclusions. Therefore, the efficacy results presented here are for the 'intention to treat population' since this more closely reflects clinical practice.

### *Acute nausea and emesis*

Pre-treatment nausea was absent in 94% of the patients, 5% of the patients had mild nausea. After 8 h of study treatment 88% (I), 87% (II), and 85% (III) of the patients had none or mild nausea. The percentage of patients experiencing none or mild nausea after 24 h were 77% in group I and 75% in groups II and III ($P > 0.5$). The results are shown in Figure 1.

**Table I** Patient demography

| | Number of patients (%) 8 mg + 1 mg h$^{-1}$ | 32 mg | 8 mg | Total |
|---|---|---|---|---|
| Patients randomised | 182 | 180 | 173 | 535 |
| Sex | | | | |
| Male | 82 (45) | 95 (53) | 86 (50) | 263 (49) |
| Female | 100 (55) | 85 (47) | 87 (50) | 272 (51) |
| Age (years) | | | | |
| 19–29 | 10 (5) | 12 (7) | 5 (3) | 27 (5) |
| 30–65 | 136 (75) | 117 (65) | 120 (69) | 373 (70) |
| >65 | 36 (20) | 51 (28) | 48 (28) | 135 (25) |
| Median | 57.5 | 60 | 60 | 59 |
| Range | 19.84 | 19.77 | 25.82 | 19.84 |
| Primary tumour site | | | | |
| Head and neck | 31 (17) | 30 (17) | 27 (16) | 88 (16) |
| Lung | 30 (16) | 41 (23) | 39 (23) | 110 (21) |
| Gastrointestinal | 15 (8) | 10 (6) | 9 (5) | 34 (6) |
| Genitourinary | 28 (15) | 22 (12) | 25 (15) | 75 (14) |
| Gynaecological | 67 (38) | 66 (37) | 65 (38) | 200 (37) |
| Bone/soft tissue | 3 (2) | 3 (2) | 4 (2) | 10 (2) |
| Miscellaneous | 11 (4) | 13 (3) | 11 (1) | 35 (4) |
| Alcohol intake | | | | |
| None of <7/week | 143 (79) | 40 (78) | 132 (76) | 415 (78) |
| 1–4 u/day | 25 (14) | 25 (14) | 27 (16) | 77 (14) |
| >4 u/day | 14 (8) | 14 (8)) | 13 (8) | 41 (8) |

1 unit of alcohol = one measure of spirit, one glass of wine or 250 ml of beer.

**Figure 1** Control of acute nausea with the continuous infusion ($n$ = 182), 32 mg single dose ($n$ = 17) schedules: nausea graded as none ▧; mild ▨; moderate ▨; severe □ at 8 and 24 h after cisplatin administration.

Results for the control of acute emesis are shown in Figure 2. Complete and major responses were achieved in 74% (Group I), 78% (Group II) and 74% (Group III) of patients. In the pairwise treatment comparisons, there were no statistically significant differences between the three dose regimens. The pattern of emesis, expressed as the total number of episodes occurring at hourly intervals over 24 h was similar in the three groups of patients (Figure 3).

Fifty two per cent of patients in group I, 53% in group II and 51% in group III had no emesis and reported none or mild nausea over the 24 h period.

*Influence of cisplatin dose and concomitant chemotherapy*

A retrospective stratification of efficacy data (emesis data) on the basis of the doses of cisplatin administered and concurrent treatment with other cytotoxic agents revealed that there were no statistically significant differences between the treatment groups for these prognostic factors. Stratification of the pooled data is shown in Table III. Overall, complete control of emesis was achieved in a significantly greater proportion of patients (157/242, 65%) who received cisplatin at doses <70 mg m$^{-2}$ compared with 137 of 293 (48%) patients who received higher doses of cisplatin (⩾70 mg m$^{-2}$; $P$ <0.001). Of the 107 patients who received cisplatin at doses ⩾100 mg m$^{-2}$, complete control was achieved at 16 or 34 (47%), 21 of 46 (46%). and 11 of 27 (41%) of patients in Groups I, II, and III respectively. The concurrent use of other moderately emetogenic agents also significantly affected the degree of control of emesis; complete control was achieved in 114 of 167 (68%) patients who received cisplatin alone, compared with 84 of 190 (44%) patients who received other emetogenic cytotoxic agents concurrently ($P$ <0.001).

*Influence of patient gender*

A retrospective stratification of the efficacy data on the basis of patient gender revealed that there were no statistically significant differences between the treatment groups for this factor. However, stratification of the pooled efficacy data as shown in Tables III and IV indicated that overall, complete control of emesis was achieved in a significantly higher proportion of male patients (67% *vs* 43%, $P$ <0.001). The observed difference was not influenced by the doses of cis-

**Table II** Concurrent chemotherapy and cisplatin dose

| | Number of patients (%) 8 mg + 1 mg h$^{-1}$ | 32 mg | 8 mg | Total |
|---|---|---|---|---|
| Patients randomised | 182 | 180 | 173 | 535 |
| Concurrent chemotherapy | | | | |
| None | 58 | 57 | 63 | 178 |
| Cyclo/ifosfamide | 32 | 37 | 36 | 105 |
| Epi/doxorubicin | 17 | 14 | 11 | 42 |
| Cyclphosph/epi/ doxorubicin | 13 | 8 | 11 | 32 |
| Eto/teniposide | 18 | 21 | 19 | 58 |
| 5-Fluorouracil | 16 | 17 | 14 | 47 |
| Miscellaneous[a] | 28 | 26 | 19 | 73 |
| Cisplatin dose | | | | |
| <50 mg m$^{-2}$ | 11 (6) | 6 (3) | 10 (6) | 27 (5) |
| 50–69.9 mg m$^{-2}$ | 79 (43) | 66 (37) | 70 (40) | 215 (40) |
| 70–99.9 mg m$^{-2}$ | 58 (32) | 62 (34) | 66 (38) | 186 (35) |
| ⩾100 mg m$^{-2}$ | 34 (19) | 46 (26) | 27 (16) | 107 (20) |
| Median dose (mg m$^{-2}$) | 70 | 76 | 71 | 72 |
| Range | 30–125 | 31–124 | 37–153 | 30–153 |
| Mean administration time (h) | 2.63 | 2.33 | 2.43 | 2.46 |

[a]Miscellaneous: bleomycin, vincristine, vinblastine, vindesine, methotrexate, mitoxanthrone, mitomycin, dacarbazine.

**Figure 2** Control of acute emesis with the continuous infusion ($n$ = 182), 32 mg single dose ($n$ = 180) and 8 mg single dose ($n$ = 173) schedules: complete control ▧; major control ▨; minor control ▨; failure □.

**Figure 3** Episodes of emesis during the 24 h after cisplatin administration with the continuous infusion (●—●), 32 mg single dose (---) and 8 mg single dose (....) schedules.

**Table III** Proportions of patients with complete responses stratified on the basis of patient gender, cisplatin dose and concomitant chemotherapy

| *Prognostic factor* | *Total number of patients (%)*[a] |
|---|---|
| Patient | |
| Male | 177/263 (67%) |
| Female | 117/272 (43%) |
| Cisplatin dose | |
| $<70$ mg$^{-2}$ | 157/242 (65%) |
| 70–99 ng m$^{-2}$ | 90/186 (48%) |
| $\geqslant 100$ mg m$^{-2}$ | 47/107 (44%) |
| Concomitant chemotherapy | |
| None | 114/167 (68%) |
| Mildly emetogenic | 96/178 (54%) |
| Moderately emetogenic | 84/190 (44%) |

[a]Pooled data; the differences were consistent within each treatment group. Concomitant chemotherapy: moderately emetogenic: cyclophosphamide, ifosfamide, epi/doxorubicin, dacarbazine; mildly emetogenic: 5-fluorouracil, mitoxanthrone, mitomycin, bleomycin, etoposide, vinblastin, vincristine.

platin or concurrent cytotoxic agents administered to the patients.

### *Adverse events*

All three dosage schedules were well tolerated; in particular, the 32 mg single dose was not associated with an increase in the incidence of adverse events. The most commonly reported events considered by the investigator to be possibly, probably or almost certainly related to ondansetron are listed in Table

**Table IV** Proportions of male and female patients with complete responses, stratified on the basis of cisplatin dose and concomitant chemotherapy

| | *Number of patients (%)* | |
|---|---|---|
| | *Male* | *Female* |
| Cisplatin dose | | |
| $<70$ mg m$^{-2}$ | 89/114 (78) | 68/128 (53) |
| 70–99 mg m$^{-2}$ | 53/84 (63) | 37/102 (36) |
| $\geqslant 100$ mg m$^{-2}$ | 35/63 (56) | 13/44 (27) |
| Concomitant chemotherapy | | |
| None | 84/109 (77) | 30/58 (52) |
| Mildly emetogenic | 71/123 (58) | 25/55 (45) |
| Moderately emetogenic | 22/31 (71) | 62/159 (39) |

Concomitant chemotherapy: moderately emetogenic: cyclophosphamide, ifosfamide, epi/doxorubicin, dacarbazine; mildly emetogenic: 5-fluorouracil, mitoxantrone, mitomycin, bleomycin, etoposide, vinblastin, vincristine.

V. Headache was the most commonly reported adverse event (11% of patients). None of these patients were withdrawn from the study and the symptoms resolved spontaneously or were treated with mild analgesics. Two major adverse events were considered to be possibly related to ondansetron treatment: one case of severe constipation and one case of pseudomembranous colitis, which resolved spontaneously. Transient changes in ALT/AST which were considered to be related to ondansetron, occurred in four patients of group I, in seven patients of group II and in two patients of group III. All changes resolved at follow-up, and none were associated with any clinical signs or symptoms.

### Discussion

Several studies have shown ondansetron to be a safe and efficacious anti-emetic in the prevention of cisplatin-induced emesis. Pharmacokinetic modelling suggested that ondansetron given as an 8 mg intravenous loading dose followed by 1 mg h$^{-1}$ for 24 h would give consistent plasma levels of 30 ng ml$^{-1}$. These levels were considered to be optimal for blocking $5HT_3$ receptors and maximising anti-emetic efficacy. Two comparative trials which investigated the efficacy of this selected dosing schedule (de Mulder *et al.*, 1990; Marty *et al.*, 1990) showed ondansetron to be superior to high-dose metoclopramide in the prophylaxis of acute cisplatin-induced emesis. This trial has investigated whether single prophylactic doses of ondansetron are as effective as the constant infusion schedule and the contribution of the 24 h continuous infusion to overall efficacy. Single dose prophylaxis would have obvious benefits to patients and hospital staff alike, and in addition, lower effective doses would reduce the cost of treatment.

The most striking observation in this study is the similarity in anti-emetic control achieved with the three treatment schedules, either for complete and/or major response (approximately 75% of patients) as well as for the control of emesis and nausea considered together (approximately 52% of patients). These results are consistent with two other comparative trials that investigated the efficacy of the con-

**Table V** Adverse events

| *Adverse event* | *Number of patients (%)* 8 mg + 1 mg h$^{-1}$ ($n$ = 182) | 32 mg ($n$ = 180) | 8 mg ($n$ = 173) | *Total* ($n$ = 535) |
|---|---|---|---|---|
| Headache | 16 (9) | 25 (14) | 20 (12) | 61 (11) |
| Diarrhoea | 3 (2) | 5 (3) | 5 (3) | 13 (2.5) |
| Constipation | 3 (2) | 3 (2) | – | 6 (1) |
| Flushing | 2 (1) | 2 (1) | – | 4 (0.8) |
| Xerostomia | 1 (0.5) | 3 (2) | – | 4 (0.8) |
| Laboratory changes | 4 (2) | 7 (4) | 2 (1) | 13 (2.5) |
| Miscellaneous | 11 (6) | 14 (8) | 10 (6) | 35 (7) |

tinuous infusion regimen of ondansetron (de Mulder *et al.*, 1990; Marty *et al.*, 1990), and a recent trial where complete control of emesis was reported in 58% of patients with a single intravenous dose of 32 mg and in 57% with the continuous infusion schedule (Marty & d'Allens, 1990).

The patterns of emesis over the 24 h period in patients who experienced emesis provide further evidence that the three dose schedules are equally efficacious. The half-life of elimination of ondansetron is approximately 3.5 h in healthy volunteers (Blackwell & Harding, 1989) and young patients (Lazarus *et al.*, 1990) but may be up to 7 h in elderly patients (Priestman *et al.*, 1990). Following a single bolus dose of 8 mg of ondansetron, plasma levels fall to below 5 ng $ml^{-1}$ at 12 h, compared to consistent levels of 30–50 ng $ml^{-1}$ with the continuous infusion schedule used in this study (Colthup & Palmer, 1989; Seynaeve *et al.*, 1990). The similar degree of anti-emetic control and pattern of emesis experienced by patients in the three treatment groups indicates that the constant plasma levels afforded by the continuous infusion regimen confer no additional benefit during the acute phase of emesis. This emphasises that the period up to 12 h following the cisplatin infusion may be the critical period for acute anti-emetic control. During this period, elevations in urinary levels of 5-HIAA, a urinary metabolite of 5HT, have been observed (Cubeddu *et al.*, 1990). The plasma levels afforded by the 8 mg single dose are probably adequate for antagonising 5HT-mediated emesis at $5HT_3$ receptors, providing protection in the majority of patients. Continuous antagonism at $5HT_3$ receptors in the 24 h following cisplatin may not be necessary for conferring any additional benefit, hence the similar efficacies observed with the 8 mg single dose and constant infusion schedules.

Several prognostic factors (Tonato *et al.*, 1991) such as previous exposure to chemotherapy, patient age, gender, chronic alcohol use, and dose of cisplatin administered are known to affect the control of chemotherapy-induced nausea and vomiting. This large parallel group study was designed to include chemotherapy-naive patients only and all the important prognostic factors were well balanced within the three groups. The comparable efficacy observed with the 8 mg single dose, in particular, cannot therefore be attributed to a chance selection of patients who were likely to have a more favourable response into this treatment group.

Some interesting points emerged from the retrospective stratifications of response based on gender and the concurrent use of cytotoxic agents. It is known that emesis in women is more difficult to control than in men (Tonato *et al.*, 1991), but it is not clear whether this is due to an underlying mechanism(s) or the more frequent use of moderately emetogenic agents such as cyclosphosphamide or doxorubicin with cisplatin in women. In this study, the degree of control of emesis (complete response) was significantly lower in female patients. This difference was consistently observed in further retrospective stratifications to determine the effect of cisplatin dose or concurrent chemotherapy on treatment outcome in men and women. Our results suggest that although the use of concurrent cytotoxics affect treatment outcome in women, they are not an influencing factor on their own and that other factor(s) therefore may be involved. Humoral factors (Carl *et al.*, 1989) are unlikely to explain the observed differences between men and women. Whole blood and plasma 5-HT levels are higher in healthy women than men but no data are available on the fluctuation in levels of the neurotransmitter in patients of different gender receiving chemotherapy (Ortiz *et al.*, 1988). It is known that anticipatory nausea and vomiting in chemotherapy-induced emesis are associated with a susceptibility to motion sickness and anxiety in addition to other characteristics (Morrow & Dobkin, 1988). It may also be that these factors are particularly relevant to women in the control of chemotherapy-induced emesis. Further attempts to elicit the physiological mechanism should be encouraged. Moreover, further studies should utilise prospective stratifications based on patient gender and cisplatin doses and include a pre-trial history about anxiety, motion sickness and vomiting during pregnancy (Martin & Diaz-Rubio, 1990) to determine the effect of these factors on treatment outcome and to optimise the most suitable prophylactic anti-emetic regimens for women.

In the population studied, the majority of patients (80%) received cisplatin at doses $<100$ mg $m^{-2}$ and the continuous infusion of 1 mg $h^{-1}$ or a higher single dose of 32 mg conferred no additional benefits over a single 8 mg dose. It is known that the degree of emesis experienced by cisplatin-treated patients is related to the dose of cisplatin administered (Tonato *et al.*, 1991) and complete control of emesis was achieved in a significantly lower proportion of the 107 patients who received cisplatin at doses $\geqslant 100$ mg $m^{-2}$. Within this group of 107 patients (20% of patients) there were no statistically differences in response rates between the three treatment schedules. However, the power of the comparisons was lower than that carried out for the response rates between treatment groups for patients who received cisplatin at doses $<70$ mg $m^{-2}$.

Although serotonin is a significant mediator of acute emesis (Cubeddu *et al.*, 1990), failure to completely protect all patients indicates that other mechanism(s) may also be involved. The addition of dexamethasone to ondansetron has been shown to significantly improve anti-emetic control (Roila *et al.*, 1991). As the mechanism and site of action of dexamethasone are not yet known, it is possible that dexamethasone contributes to overall efficacy by suppressing one or more of these additional mechanism(s).

The adverse events considered to be related to ondansetron were generally mild in nature, and the incidences were similar between the treatment schedules. As previously observed, headache was the most common event.

In conclusion, this study shows that a single intravenous dose of 8 mg of ondansetron is as efficacious as a 32 mg daily dose in the prophylaxis of acute cisplatin-induced emesis. In the population studied, a continuous infusion of 1 mg/hour for 24 h conferred no additional benefit in anti-emetic protection. The efficacy of single dose anti-emetic prophylaxis is likely to improve patient and nursing staff acceptance of ondansetron; moreover, it should allow out-patient treatment where appropriate.

We wish to thank the nurses in the different centres who were involved with the recording of data and Dr J. Verweij for advice in preparation of the manuscript.

### References

BLACKWELL, C. & HARDING, S.M. (1989). The clinical pharmacology of ondansetron. *Eur. J. Can. Clin. Oncol.*, **25**, Suppl 1, S21–S24.

CARL, P.L., CUBEDDU, L.X., LINDLEY, C., MYERS, R.D. & REZVANI, A.H. (1989). Do humoral factors mediate cancer chemotherapy-induced emesis? *Drug Metab. Rev.*, **21**, 21, 319–333.

CLARK, R.A., KRIS, M.G., GRALLA, R.J. & TYSON, L.B. (1990). Serotonin antagonists demonstrate antiemetic effectiveness without extrapyramidal symptoms. Analysis of studies with three agents. *Proc. Amer. Soc. Clin. Oncol.*, **9**, 322.

COLTHUP, P.V. & PALMER, J. (1989). The determination in plasma and pharmacokinetics of ondansetron. *Eur. J. Cancer Clin. Oncol.*, **25**, S71–S74.

CUBEDDU, L.X., HOFFMANN, I.S., FUENMAYOR, N.T. & FINN, A.L. (1990). Efficacy of andansetron and the role of serotonin in cisplatin-induced nausea and vomiting. *N. Engl. J. Med.*, **322**, 810–816.

DE MULDER, P.H.M., SEYNAEVE, C., VERMORKEN, J.B. & 5 others (1990). Ondansetron versus high-dose metoclopramide in the prophylaxis of acute and delayed cisplatin-induced nausea and vomiting. *Ann. Int. Med.*, **113**, 834–840.

GRALLA, R.J., ITRI, L.M., PISKO, S.E. & 6 others (1981). Antiemetic efficacy of high-dose metoclopramide: randomized trials with placebo and prochlorperazine in patients with chemotherapy-induced nausea and vomiting. *N. Engl. J. Med.*, **305**, 905–909.

HAINSWORTH, J., HARVEY, W., PENDERGRASS, K. & 10 others (1991). A single-blind comparison of intravenous ondansetron, a selective serotonin antagonist, with intravenous metoclopramide in the prevention of nausea and vomiting associated with high-dose cisplatin chemotherapy. *J. Clin. Oncol.*, **9**, 721–728.

KRIS, M.G., GRALLA, R.J., CLARK, R.A., TYSON, L.B. & GROSHEN, S. (1987). Antiemetic control and prevention of side effects of anticancer therapy with lorazepam or diphenhydramine when used in combination with metoclopramide plus dexamethasone. *Cancer*, **60**, 2816–2822.

LAZARUS, H.M., BRYSON, K.C., LEMON, E., PRITCHARD, J.F. & BLUMER, J. (1990). Antiemetic efficacy and pharmacokinetic analyses of ondansetron during multiple-day chemotherapy with cisplatin prior to autologous bone marrow transplantation. *J. Natl Cancer Inst.*, **82**, 1776–1778.

MARTIN, M. & DIAZ-RUBIO, E. (1990). Emesis during pregnancy: a new factor in chemotherapy-induced emesis. *Annals. Oncol.*, **1**, 152–153.

MARTY, M., POUILLART, P., SCHOLL, S. & 7 others (1990). Comparison of the serotonin antagonist ondansetron with high-dose metoclopramide in the control of cisplatin-induced emesis. *N. Engl. J. Med.*, **322**, 816–821.

MARTY, M. & D'ALLENS, H. (1990). Etude randomisèe en double-insu comparant l'efficacité de l'ondansetron selon deux modes d'administration: injection unique et perfusion continue. *Cahiers Cancer*, **2**, 541–546.

MORROW, G.R. & DOBKIN, P.L. (1988). Anticipatory nausea and vomiting in cancer patients undergoing chemotherapy treatment: prevalence, etiology and behavioral interventions. *Clin. Psychol. Rev.*, **8**, 517–556.

ORTIZ, J., ORTIGAS, F. & FELPI, E. (1988). Serotonergic status in human blood. *Life Sci.*, **43**, 983–990.

PRIESTMAN, T.J., UPADHYAYA, B.K., PALMER, J.L. & COLTHUP, P.V. (1990). Pharmacokinetics of the antiemetic, ondansetron. *Ann. Oncol.*, **1**(S), 114.

ROILA, F., TONATO, M., BASURTO, C. & 10 others (1989). Protection from nausea and vomiting in cisplatin-treated patients: high-dose metoclopramide combined with methylprednisolone versus metoclopramide combined with dexamethasone and diphenhydramine. *J. Clin. Oncol.*, **7**, 1693–1700.

ROILA, F., TONATO, M., COGNETTI, F. & 9 others (1990). Prevention of cisplatin-induced emesis: a double-blind multicentre randomised crossover study comparing ondansetron and ondansetron plus dexamethasone. *J. Clin. Oncol.*, **9**, 675–678.

ROILA, F., BASURTO, C., BRACARDA, S. & 6 others (1991). Double-blind crossover trial of single versus divided dose of metoclopramide in a combined regimen for treatment of cisplatin-induced emesis. *Eur. J. Can.*, **27**, 119–121.

SEYNAEVE, C., DE MULDER, P.H.M., VAN LIESSUM, P., LANE-ALLMAN, E., SCHMITZ, P. & VERWEIJ, J. (1990). A positive correlation of the plasma ondansetron level with the control of cisplatin-induced emesis. *Ann. Oncol.*, **1**(S), 112

SORBE, B., FRANKENDAL, B., GLIMELIUS, B., HANSEN, O. & PRUEMM, V. (1990). A multicentre randomised study comparing the antiemetic effects of the 5-$HT_3$ antagonist ICS205-930 with a metoclopramide containing antimetic cocktail in patients receiving cisplatin chemotherapy. *Ann. Oncol.*, **1**(S), 113.

SOUKOP, M. (1990). A comparison of two dose levels of granisetron in patients receiving high-dose cisplatin. *Eur. J. Cancer*, **26**(S1), 15–19.

TONATO, M., ROILA, F. & DEL FAVERO, A. (1991). Methodology of antiemetic trials: a review. *Ann. Oncol.*, **2**, 107–114.

VAN ELTEREN, P.H. (1960). On the combination of independent two sample tests of Wilcoxon. *Bull. Int. Statist. Inst.*, **37**, 351–361.